# Improved quality of life and psychological symptoms following mindfulness and cognitive rehabilitation in multiple sclerosis and their mediating role for cognition: a randomized controlled trial

**DOI:** 10.1007/s00415-024-12327-y

**Published:** 2024-04-23

**Authors:** Ilse M. Nauta, Maureen van Dam, Dirk Bertens, Roy P. C. Kessels, Luciano Fasotti, Bernard M. J. Uitdehaag, Anne E. M. Speckens, Brigit A. de Jong

**Affiliations:** 1 Department of Neurology, MS Center Amsterdam, Vrije Universiteit Amsterdam, Amsterdam Neuroscience, Amsterdam UMC Location VUmc, Amsterdam, The Netherlands; 2 Department of Anatomy and Neurosciences, MS Center Amsterdam, Vrije Universiteit Amsterdam, Amsterdam Neuroscience, Amsterdam UMC Location VUmc, PO Box 7057, 1007 MB Amsterdam, The Netherlands; 3 Institute of Psychology, Health, Medical and Neuropsychology Unit, Leiden University, Wassenaarseweg 52, Leiden, The Netherlands; 4 Donders Institute for Brain, Cognition and Behaviour, Radboud University, Nijmegen, The Netherlands; 5 Klimmendaal Rehabilitation Center, Arnhem, The Netherlands; 6 Vincent Van Gogh Institute for Psychiatry, Venray, The Netherlands; 7 Department of Medical Psychology, Radboud University Medical Center, Nijmegen, The Netherlands; 8 Department of Psychiatry, Radboud University Medical Center, Nijmegen, The Netherlands

**Keywords:** Multiple sclerosis, Mindfulness, Cognitive rehabilitation, Quality of life, Depressive and anxiety symptoms

## Abstract

**Background:**

Multiple sclerosis (MS) frequently gives rise to depressive and anxiety symptoms, but these are often undertreated. This study investigated the effect of mindfulness-based cognitive therapy (MBCT) and cognitive rehabilitation therapy (CRT) on psychological outcomes and quality of life (QoL), and whether they mediate treatment effects on MS-related cognitive problems.

**Methods:**

This randomized controlled trial included MS patients with cognitive complaints (*n* = 99) and compared MBCT (*n* = 32) and CRT (*n* = 32) to enhanced treatment as usual (*n* = 35). Baseline, post-treatment and 6-months follow-up assessments included patient-reported outcome measures (PROMS) and cognitive outcomes (self-reported and neuropsychological assessment). PROMS concerned psychological symptoms, well-being, QoL, and daily life function. Linear mixed models indicated intervention effects on PROMS and mediation effects of PROMS on cognitive outcomes.

**Results:**

MBCT positively affected depressive symptoms (Cohen’s *d* (*d*) = −0.46), fatigue (*d* = −0.39), brooding (*d* = −0.34), mindfulness skills (*d* = 0.49), and mental QoL (*d* = −0.73) at post-treatment. Effects on mindfulness skills remained significant 6 months later (*d* = 0.42). CRT positively affected depressive symptoms (*d* = −0.46), mindfulness skills (*d* = 0.37), and mental QoL (*d* = −0.45) at post-treatment, but not at 6-month follow-up. No effects on anxiety, well-being, self-compassion, physical QoL, and daily life function were found. Treatment effects on self-reported, but not objective, cognition were mediated by psychological symptoms and mindfulness skills.

**Conclusions:**

MBCT and CRT reduced a wide array of psychological symptoms and improved mental QoL. These improvements seemed to impact self-reported cognitive problems after both treatments, whereas objective cognitive improvements after MBCT seemed independent of improvement in psychological symptoms. Future studies should investigate long-term sustainability of these beneficial effects.

**Trial registration:**

The trial was prospectively registered in the Dutch Trial registry on 31 May 2017 (NL6285; https://trialsearch.who.int/Trial2.aspx?TrialID=NTR6459).

**Supplementary Information:**

The online version contains supplementary material available at 10.1007/s00415-024-12327-y.

## Introduction

Multiple sclerosis (MS) is the most frequent demyelinating and neurodegenerative disease of the central nervous system in young adults [1]. Due to the unpredictable nature of symptoms in MS in terms of their frequency, severity, and trajectory, comorbid psychological symptoms are often present [2]. Patients report a significantly higher prevalence of severe fatigue [3], cognitive complaints [4], and clinical depression [5] compared to the general population [6]. This frequently leads to a reduced quality of life (QoL) [7] and poorer adherence to disease-modifying therapies [8]. To manage these psychological symptoms, it is particularly important for treatment effects to impact on multiple psychological symptoms, given their complex interrelationship [9].

Recent advances in the field have shown promising effects of mindfulness-based interventions on psychological symptoms in MS patients [10], especially with regard to depressive symptoms [11], fatigue [12], and QoL [13]. Preliminary beneficial effects have also been reported for cognition [14]. With mindfulness-based interventions, the goal is to increase awareness of the present moment, which is suggested to help the patient to respond more attentively to disease-related challenges [11]. As for cognition, it is unclear whether the preliminary effects on cognitive impairments and self-reported cognitive complaints are a result of improvements in psychological symptoms [14], given their interrelationship [9], or whether mindfulness is able to affect cognition independently. Importantly, cognitively impaired patients were mostly excluded from previous studies [13], but given the high prevalence of cognitive impairment in MS (up to 65%) [15], it is essential to include them in investigating whether mindfulness-based interventions are effective in reducing psychological symptoms.

Another promising intervention is cognitive rehabilitation therapy (CRT), targeted at the compensation of cognitive impairments (i.e., through the use of strategies) or restoring cognitive functions [16, 17]. In particular with compensatory CRT, where the aim is to improve patients’ function in daily life, it may be expected that cognitive effects could coincide with improvements in psychological symptoms. However, previous studies mainly focused on objective cognitive test results and rarely took other psychological outcomes into account, although there were some indications that QoL could be improved [18–20]. The few studies that investigated depressive symptoms after CRT have yielded mixed results [18–20].

Therefore, the aim of the present study was to establish the effectiveness of both mindfulness-based cognitive therapy (MBCT) and compensatory CRT on psychological symptoms, QoL, well-being, and daily life function in MS patients with cognitive complaints. Hence, we compared the REMIND-MS cohort [i.e., a longitudinal, single-blind, randomized controlled trial (RCT) with a control group that received enhanced treatment as usual (ETAU)] [21]. The outcome measures entailed the secondary outcome measures of the REMIND-MS trial [21]. The effects on the primary outcome measure, i.e., the level of self-reported cognitive complaints, and objective cognition have been reported before [14]. Secondly, we addressed the interrelationship between treatment effects on psychological symptoms and the previously published positive effects on cognitive problems [14], including self-reported cognitive complaints, personalized cognitive goals, and information processing speed (i.e., the most affected cognitive function in MS) [4].

## Methods

### Participants and study design

**Fig. 1:**
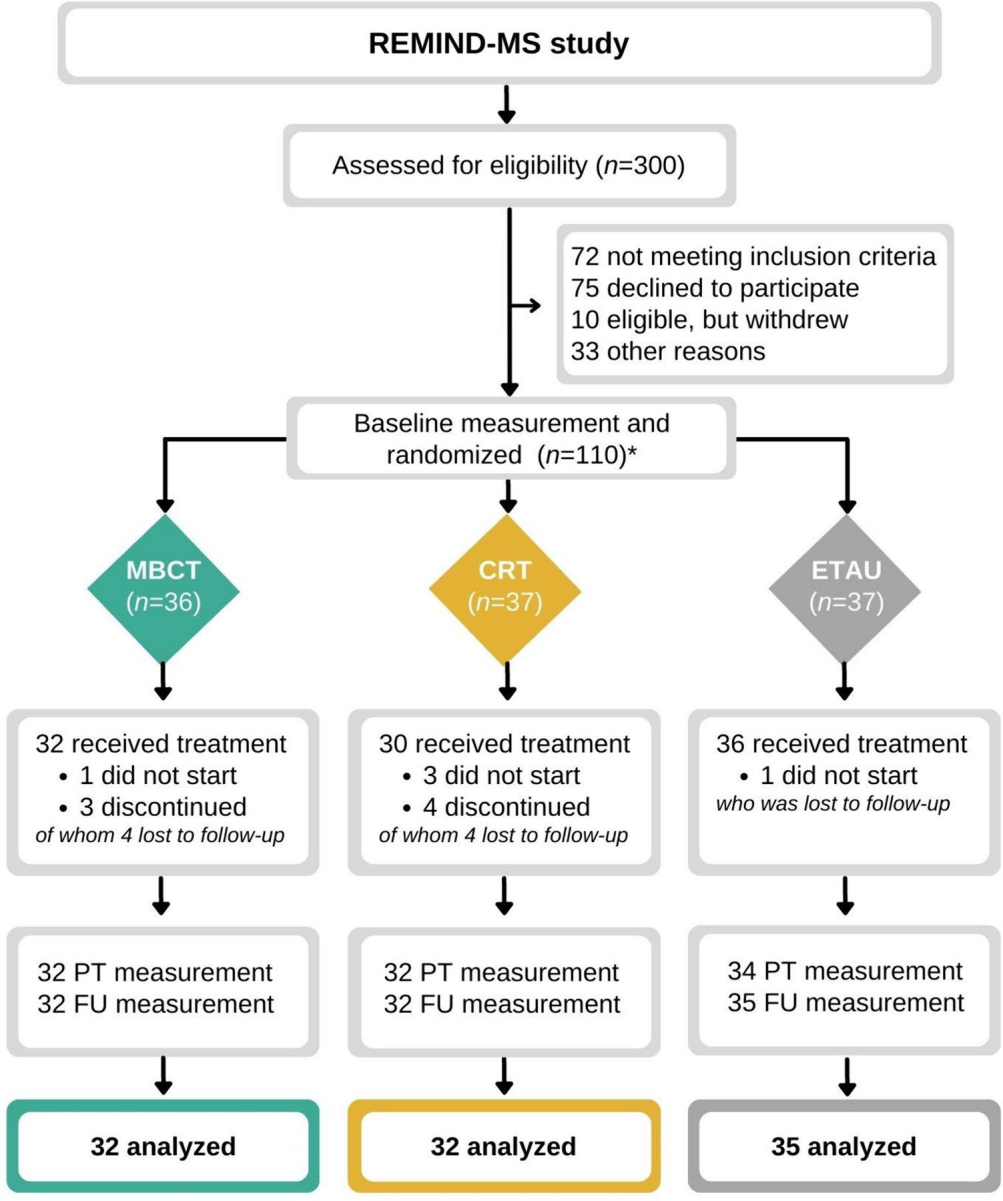
Flowchart REMIND-MS study. *One patient randomized into the ETAU group did not complete baseline questionnaires, and could therefore not be included in our analyses that concerned questionnaires, leading to 35 analyzed patients in the ETAU group. *CRT* cognitive rehabilitation therapy; *MBCT* mindfulness-based cognitive therapy; *ETAU* enhanced treatment as usual; *PT* post-treatment; *FU* 6-month follow-up

The REMIND-MS study is a longitudinal dual-center, single-blind RCT with three parallel groups: MBCT, CRT and ETAU. A detailed overview of the study protocol, full eligibility criteria, and randomization procedure has been published elsewhere [14, 21]. In short, study measurements included a baseline, post-treatment and 6-month follow-up assessment, collected between December 2017 and November 2020 (see Fig. 1 for the flowchart of the study design). Following baseline assessment, random allocation was performed per treatment location (i.e., Amsterdam MS Center and Klimmendaal Rehabilitation Center Arnhem) to MBCT, CRT, or ETAU (blocks varied between 6 and 9; 1:1:1 ratio), and assessors were blind to treatment allocation. Inclusion criteria included (1) verified MS diagnosis (McDonald 2010 criteria) [22], (2) 18–65 years of age, (3) cognitive complaints (scoring ≥ 23 on the Multiple Sclerosis Neuropsychological Questionnaire-Patient version (MSNQ-P) [23], and (4) no previous experience with the interventions.

Medical ethical approval was obtained from the institutional ethics review board of the Amsterdam UMC (2017.009). All patients provided written informed consent prior to inclusion. The procedures in this study comply with the ethical standards of the relevant national and institutional committees on human experimentation and with the Helsinki Declaration of 1975, as revised in 2008.

### Interventions

In this RCT, the control group (ETAU) had an appointment with an MS specialist nurse, focused on psychoeducation (with the same information as given during MBCT and CRT). MBCT entailed a standard 8-week MBCT protocol, performed during nine weeks (i.e., eight weekly 2.5-h sessions, and a 5-h silent retreat performed in a separate week). During MBCT, patients were trained to self-regulate their attention and to be aware of moment-to-moment experiences (e.g., emotions, thoughts, and behaviors) in a non-judgmental manner [24]. CRT also lasted nine weeks, including nine weekly 2.5-h sessions. This treatment focused on the learning and application of compensatory strategies to problems in processing speed [25], memory [26], executive function [27], and mental fatigue [28]. It also addressed emotional and behavioral changes and grief resolution. MBCT and CRT were group-based treatments (MBCT 4–7 patients, CRT 3–6 patients). Patients received meditation exercises (for MBCT) and homework assignments (for CRT) lasting 30–45 min/day for 6 days/week. Patient’s adherence was documented, and therapists’ competence and protocol adherence were evaluated based on video and audio recordings of MBCT and CRT sessions, respectively [29].

### Patient-reported outcome measures

Information on demographics and disease-related characteristics (e.g., type of MS and disease duration) were collected at baseline. Patient-reported outcome measures (PROMS) reported hereafter were collected at all measurement points.

#### Psychological symptoms

Symptoms of anxiety and depression were measured using the Hospital Anxiety and Depression Scale (HADS) [30]. The level of fatigue was assessed with the Fatigue severity subscale of the Checklist Individual Strength (CIS-20) [31]. The tendency to ruminate when being sad or depressed was measured with the ‘Brooding’ subscale of the Dutch Ruminative Response Scale (RRS-NL) [32].

#### Quality of life

Health-related QoL was measured with the Multiple Sclerosis Quality of Life Questionnaire (MSQoL-54) and included two subscales: the Physical Health Composite score (PHC) and the Mental Health Composite score (MHC) [33].

#### Daily life functioning

Participation in societal activities was measured with the Utrecht Scale for Evaluation of Rehabilitation-Participation (USER-P) [34].

#### Well-being

Emotional, psychological, and social well-being was measured using the Mental Health Continuum-Short Form (MHC-SF) [35].

#### Mindfulness skills

The ability to be mindful, i.e., non-judgmental aware of moment-to-moment experiences, was assessed with the Five Facets of the Mindfulness Questionnaire Short Form (FFMQ-SF) [36]. To measure self-compassion, i.e., the ability to act with compassion toward oneself in difficult times, the short form of the Self-Compassion Scale was used [37].

### Cognitive outcomes

The level of self-reported cognitive complaints was measured with the Cognitive Failure Questionnaire (CFQ) [38] for daily cognitive problems and the Behavior Rating Inventory of Executive Function-Adult Version (BRIEF-A) for everyday executive functioning (subscales behavioral regulation and metacognition) [39]. Two personalized goals for each patient were formulated at baseline using goal attainment scaling (GAS), on a 6-point Likert scale, addressing real-life challenges due to cognitive problems faced by the patients [40]. Objective cognitive function was measured using a neuropsychological assessment primarily based on the Minimal Assessment of Cognitive Function in MS (MACFIMS) [41], while additionally including the Stroop Color-Word Test [42]. Based on the intervention effects analyzed previously [14], only the cognitive domain information processing speed was included in this study, which was constructed by a composite score of [14]: (1) the oral version of the Symbol Digit Modalities Test (SDMT) [43] and (2) the Stroop Color-Word Test (cards I and II) [42]. Supplementary Table 1 details information on the included tests and construction of cognitive domains. To control for material-specific learning effects, alternate forms were administered for repeated measurements of the SDMT.

### Power analysis

The REMIND-MS study was powered based on the CFQ (primary outcome), the results of which have been published previously [14, 21]. Considering an alpha of 0.05, power of 0.80, intra-class correlation of 0.06, and a medium effect size, a sample size of 33 patients per group was required. Accounting for potential dropouts, the intended sample size included 40 patients per group.

### Statistical analyses

Statistical analyses were performed using SPSS 28, Stata 17, and R-Studio 4.2.1 [44]. Normality of the outcome variables and residuals of the models were assessed with histogram inspection. The MHC and HADS depression subscale were transformed using the square root and the natural logarithm, respectively, as these were positively skewed. Analyses were performed on a modified intention-to-treat sample, including all patients with at least one follow-up measurement. For all analyses, α-level was set at 0.05, or Bonferroni corrected for the number of subscales within a PROM analyzed in the primary analyses.

Linear mixed-model analyses were performed for all PROMS with time (i.e., post-treatment and 6-month follow-up) as within-subjects factor and treatment (i.e., MBCT vs. ETAU and CRT vs. ETAU) as between-subjects factor. A random intercept on subject-level was included to account for the dependency of repeated observations within patients. Treatment effects irrespective of time and at both post-treatment and 6-month follow-up (inserting group-by-time interactions; group estimates indicate intervention effects) were investigated in separate models for each PROM. Age, sex, and baseline levels of the outcome variable were included as covariates. By including the baseline measurement as a covariate, the model captures individual variability at the start of the study, thus controlling for initial individual differences and mitigating the effect of regression to the mean [45], an effect which can also occur in the ETAU group.

Secondly, we investigated whether the treatment effects on cognitive outcomes, previously published in our work [14], were mediated by treatment effects on PROMS, focusing particularly on psychological symptoms and mindfulness skills. For this purpose, we expanded the longitudinal mixed-effects models to incorporate longitudinal mediations effects [46]. We analyzed the time point for which the treatment had a significant effect on the cognitive outcome. For post-treatment effects, we included the potential mediator at post-treatment. For 6-month follow-up effects, we included the potential mediator at the same time point, and in a separate model also as a change score between baseline and post-treatment, thereby studying the order of events. The conditions of mediation were investigated [47]. In other words, all the pathways of the mediation model needed to be established: (1) the treatment should affect the cognitive outcome: for this, we repeated the linear mixed model analyses of the previous study (cognitive outcomes as dependent variables; baseline level of the outcomes, age, education, and sex as covariates) [14], while also adding the baseline level of the potential mediator; (2) the treatment should affect the potential mediator; therefore, we selected psychological and mindfulness skills outcomes that were significantly affected by the treatments; (3) the potential mediator and cognitive outcome should be related (see step 4 for the methods); and (4) the mediating effect was considered present if the first three conditions were met, and if the effect of the treatments on the cognitive outcome changed ≥ 10% when adding the mediator to the model. For the steps three and four, the same model as for the first step was performed, while including the change score of the potential mediator and the follow-up scores.

## Results

The current study analyzed 99 patients, of which 32 were randomized into MBCT, 32 into CRT, and 35 into ETAU (see Fig. 1). Of these 99 patients, 74% were women and 64% had relapsing–remitting MS. Mean age of the group was 48.8 (± 9.6) years. Other demographic, disease-related, and psychological characteristics are included in Table 1. These variables did not differ between the treatment and control groups. Information on intervention adherence is reported elsewhere [14]. In short, median attendance was 8 out of 9 sessions for both MBCT and CRT, and the median homework completion was 63% for MBCT and 86% for CRT. MBCT therapists were considered beginner (*n* = 18 analyzed patients) and proficient (*n* = 14 analyzed patients), and CRT therapists proficient (*n* = 22 analyzed patients, of whom 1 did not start and 1 discontinued CRT, see Fig. 1) and advanced (*n* = 10 analyzed patients).

**Table 1:** Demographic, disease-related, and psychological characteristics of the total sample and per treatment group

		Total sample (*n* = 99)	Treatment groups			*p*-value
			MBCT (*n* = 32)	CRT (*n* = 32)	ETAU (*n* = 35)	
Demographics						
Age (in years)	Mean (SD)	48.8 (9.6)	46.0 (10.2)	50.9 (7.8)	49.3 (10.3)	0.114
Sex	f: m (%f)	73:26 (73.7%)	23:9 (71.9%)	24:8 (75.0%)	26:9 (74.3%)	0.956
Education (high)	*n *(%)	61 (61.6%)	20 (62.5%)	20 (62.5%)	21 (60.0%)	0.971
Disease-related characteristics						
MS type (RRMS/SPMS/PPMS/unclear)	(%)	64/18/13/5	59/22/13/6	63/16/16/6	69/17/11/3	0.968
Disease duration since diagnosis (years)	Median (IQR)	8.0 (3.0–19.2)	7.0 (2.7–17.0)	9.3 (3.2–18.7)	9.3 (3.9–23.5)	0.301
EDSS	Median (range)	4.0 (2.0–7.5)	3.5 (2.0–7.0)	3.8 (2.0–6.5)	4.0 (2.5–7.5)	0.761
DMT use (yes)	*n *(%)	51 (51.5%)	18 (56.3%)	18 (56.3%)	15 (42.9%)	0.444
Comorbidities (CIRS score)	Median (range)	3.0 (3.0–9.0)	3.5 (3.0–7.0)	3.5 (3.0–7.0)	3.0 (3.0–9.0)	0.147
Cognitive impairment (yes)	*n *(%)	56 (56.6%)	20 (62.5%)	18 (56.3)	18 (51.4)	0.658
Psychological characteristics						
CIS-20 fatigue severity	Mean (SD)	39.3 (10.6)	40.8 (12.0)	38.6 (10.6)	38.5 (9.3)	0.615
Significant fatigue (≥ 27)	*n *(%)	87 (87.9%)	26 (81.3%)	29 (90.6%)	32 (91.4%)	0.375
Depressive symptoms (HADS-D score)	Median (IQR)	5.0 (3.0–7.0)	5.0 (3.0–8.0)	4.0 (3.0–7.0)	4.0 (3.0–7.0)	0.251
Significant depressive symptoms (≥ 11)	*n *(%)	10 (10.1%)	4 (12.5%)	3 (9.4%)	3 (8.6%)	0.856
Anxiety (HADS-A score)	Mean (SD)	7.7 (4.1)	8.0 (4.3)	7.3 (4.1)	7.9 (4.0)	0.734
Significant anxiety symptoms (≥ 11)	*n *(%)	18 (18.2%)	6 (18.8%)	6 (18.8%)	6 (17.1%)	0.981

Education was coded according to Verhage and categorized as low (i.e., completed average-level secondary education or lower; levels 1–5) or high (i.e., completed high level secondary education or university degree; levels 6–7). MS type: unclear indicates that the MS type could not be specified by the neurologist

*MBCT* mindfulness-based cognitive therapy; *CRT* cognitive rehabilitation therapy; *ETAU* enhanced treatment as usual; *MS* multiple sclerosis; *RR* relapsing remitting; *SP* secondary progressive; *PP* primary progressive; *EDSS* Expanded Disability Status Scale; *DMT* disease-modifying therapy; *CIRS* Cumulative Illness Rating Scale; *CIS-20* Checklist Individual Strength; *HADS-D* Hospital Anxiety and Depression Scale-depression score; *HADS-A* Hospital Anxiety and Depression Scale-anxiety score; *SD* standard deviation; *IQR* interquartile range

### Treatment effects

**Fig. 2:**
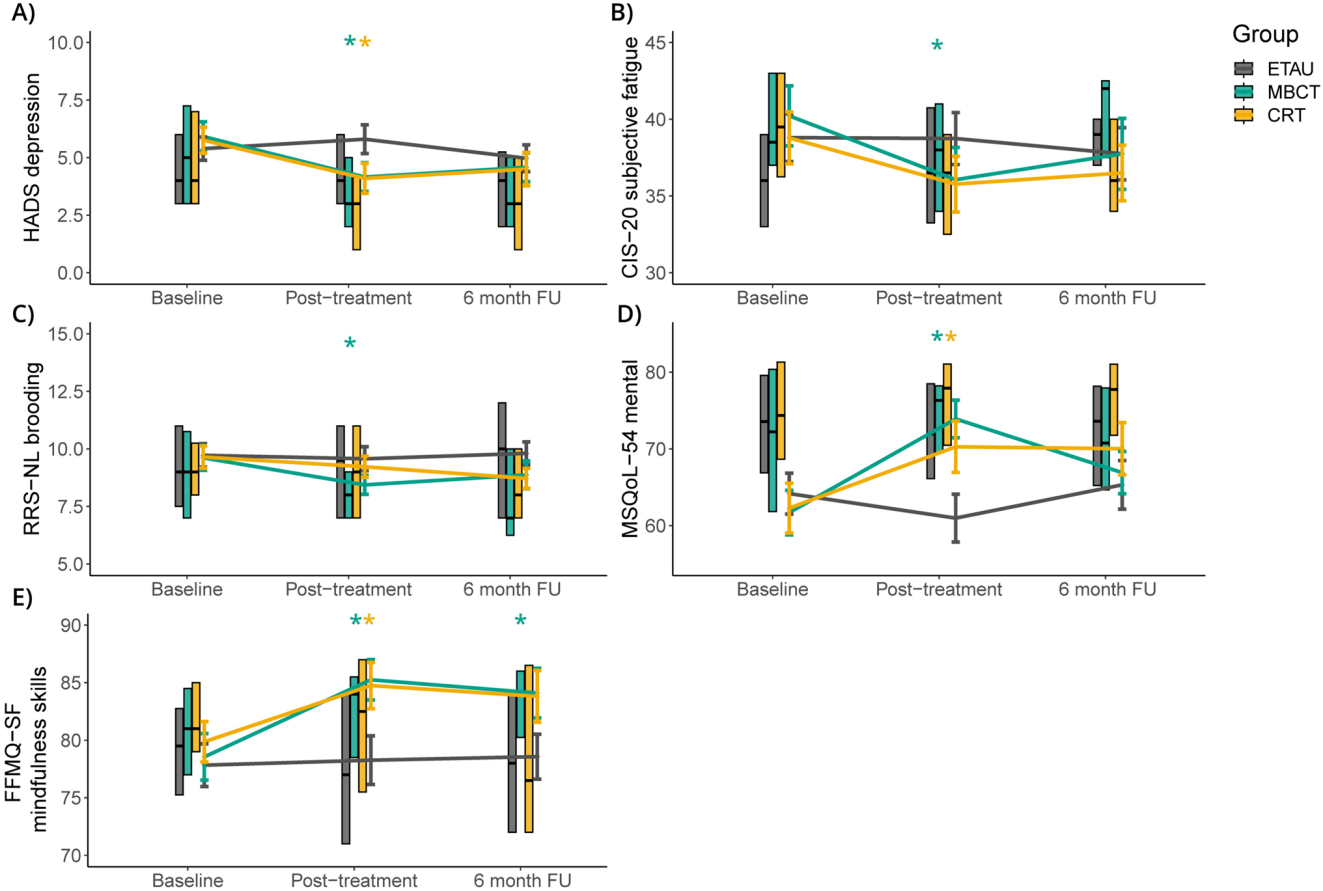
Observed intervention effects on patient-reported outcome measures. **A** Differences in HADS depression scores for the different intervention groups. **B** Differences in CIS-20 subjective fatigue scores for the different intervention groups. **C** Differences in RRS-NL brooding scores for the different intervention groups. **D** Differences in MSQoL-54 scores for the different intervention groups. **E** Differences in FFMQ-SF mindfulness skills scores for the different intervention groups. *MBCT* mindfulness-based cognitive therapy; *CRT* cognitive rehabilitation therapy; *ETAU* enhanced treatment as usual; *FU* Follow-up; *HADS* Hospital Anxiety and Depression Scale; *CIS-20* Checklist Individual Strength; *RRS-NL* Ruminative Response Scale; *MSQoL-54* Multiple Sclerosis Quality of Life Questionnaire-54; *MHC-SF* Mental Health Continuum-Short Form; *FFMQ-SF* Five Facets of the Mindfulness Questionnaire short form

Table 2 shows the intervention effects (raw scores can be found in Supplementary Table 2). Figure 2 shows only significant intervention effects. Both MBCT (β = −0.30, *p* = 0.006, Cohen’s *d* = −0.46) and CRT (β = −0.32, *p* = 0.005, Cohen’s *d* = −0.46) had a positive effect on depression at post-treatment compared to ETAU. MBCT also had a positive effect on fatigue (β = −4.31, *p* = 0.029, Cohen’s *d* = −0.39) and brooding (β = −1.01, *p* = 0.042, Cohen’s *d* = −0.34) at post-treatment. No effects were found on anxiety (*p* > 0.025). As to QoL, both MBCT (β = −1.16, *p* = 0.001, Cohen’s *d* = −0.73) and CRT (β = −0.79, *p* = 0.017, Cohen’s *d* = −0.45) had a positive effect on mental QoL at post-treatment. No effects on physical QoL were found. Mindfulness skills also improved after both MBCT (β = 5.70, *p* = 0.005, Cohen’s *d* = 0.49) and CRT (β = 4.50, *p* = 0.034, Cohen’s *d* = 0.37) at post-treatment, and this effect remained significant at 6-month follow-up after MBCT (β = 4.90, *p* = 0.017, Cohen’s *d* = 0.42). Post hoc analyses focusing on subscales of mindfulness skills showed positive effects at both time points on ‘describing’ after MBCT (*p* = 0.030 and *p* = 0.011, respectively) and CRT (*p* = 0.018 and *p* = 0.041, respectively). MBCT also had a positive effect on ‘observing’ at post-treatment (*p* = 0.003) and ‘acting with awareness’ at 6-month follow-up (*p* = 0.032). No effects on well-being (*p* > 0.05), self-compassion (*p* > 0.05), or daily life functioning were found (*p* > 0.017).

**Table 2:** Intervention effects on patient-reported outcome measures

		MBCT vs ETAU			CRT vs ETAU		
		β (95%CI)	*p*-value	Cohen’s *d*	β (95%CI)	*p*-value	Cohen’s *d*
Psychological symptoms							
HADS anxiety^a^	Overall	− 0.93 (− 2.16, 0.30)	0.137	− 0.23	− 0.56 (− 1.73, 0.62)	0.355	− 0.13
	Post-treatment	− 1.46 (− 2.89, − 0.05)	0.043^	− 0.36	− 1.15 (− 2.51, 0.22)	0.101	− 0.28
	6-month follow-up	− 0.41 (− 1.82, 1.00)	0.570	− 0.10	0.01 (− 1.34, 1.37)	0.984	0.002
HADS depression^a^ (transformed)	Overall	** − 0.19 (− 0.36, − 0.02)**	0.027*	** − 0.29**	− 0.19 (− 0.38, − 0.001)	0.049^	− 0.27
	Post-treatment	** − 0.30 (− 0.51, − 0.09)**	0.006*	** − 0.46**	** − 0.32 (− 0.55, − 0.10)**	0.005*	** − 0.46**
	6-month follow-up	− 0.08 (− 0.30, 0.13)	0.437	− 0.12	− 0.06 (− 0.28, 0.16)	0.587	− 0.09
CIS20-R fatigue severity	Overall	− 3.23 (− 6.38, − 0.07)	0.045^	− 0.29	− 2.02 (− 4.81, 0.77)	0.156	− 0.20
	Post-treatment	** − 4.31 (− 8.18, − 0.44)**	0.029*	** − 0.39**	− 2.72 (− 6.06, 0.62)	0.110	− 0.27
	6-month follow-up	− 2.14 (− 6.00, 1.72)	0.277	− 0.19	− 1.35 (− 4.65, 1.96)	0.425	− 0.13
RRS-NL brooding	Overall	** − 0.94 (− 1.75, − 0.13)**	0.022*	** − 0.31**	− 0.46 (− 1.25, 0.34)	0.261	− 0.16
	Post-treatment	** − 1.01 (− 1.98, − 0.04)**	0.042*	** − 0.34**	− 0.10 (− 1.00, 0.81)	0.836	− 0.04
	6-month follow-up	− 0.86 (− 1.83, 0.11)	0.080	− 0.29	− 0.80 (− 1.69, 0.10)	0.080	− 0.28
Quality of life							
MSQoL-54 physical^a^	Overall	1.32 (− 3.71, 6.35)	0.607	0.08	1.61 (− 2.77, 6.00)	0.471	0.10
	Post-treatment	3.54 (− 2.12, 9.20)	0.220	0.21	4.48 (− 0.48, 9.45)	0.077	0.26
	6-month follow-up	− 0.83 (− 6.48, 4.81)	0.772	− 0.05	− 1.21 (− 6.16, 3.74)	0.631	− 0.07
MSQoL-54 mental^a^ (transformed)	Overall	− 0.63 (− 1.20, − 0.07)	0.029^	− 0.40	− 0.55 (− 1.08, − 0.01)	0.047^	− 0.31
	Post-treatment	** − 1.16 (− 1.82, − 0.50)**	0.001*	** − 0.73**	** − 0.79 (− 1.43, − 0.14)**	0.017*	** − 0.45**
	6-month follow-up	− 0.10 (− 0.76, 0.56)	0.768	− 0.06	− 0.31 (− 0.95, 0.34)	0.347	− 0.17
Daily life functioning							
USER-P frequency^b^	Overall	− 2.17 (− 4.57, 0.23)	0.077	− 0.86	− 0.03 (− 2.53, 2.47)	0.980	− 0.01
	Post-treatment	− 3.13 (− 6.39, 0.13)	0.060	− 1.24	− 1.68 (− 4.83, 1.46)	0.294	− 0.69
	6-month follow-up	− 1.25 (− 4.49, 2.00)	0.452	− 0.49	1.60 (− 1.53, 4.73)	0.318	0.66
USER-P restrictions^b^	Overall	− 1.03 (− 5.54, 3.48)	0.654	− 0.30	− 0.65 (− 5.02, 3.71)	0.769	− 0.19
	Post-treatment	− 3.55 (− 8.74, 1.65)	0.180	− 1.02	− 0.87 (− 6.05, 4.32)	0.743	− 0.26
	6-month follow-up	1.46 (− 3.72, 6.64)	0.581	0.42	− 0.48 (− 5.64, 4.68)	0.855	− 0.14
USER-P satisfaction^b^	Overall	− 3.62 (− 8.51, 1.27)	0.147	− 1.67	− 2.58 (− 7.80, 2.65)	0.334	− 1.16
	Post-treatment	− 1.21 (− 7.29, 4.87)	0.696	− 0.56	− 3.31 (− 9.49, 2.87)	0.294	− 1.49
	6-month follow-up	− 6.13 (− 12.19, − 0.06)	0.048^	− 2.82	− 1.85 (− 8.00, 4.30)	0.556	− 0.83
Well-being							
MHC-SF well-being	Overall	0.21 (− 0.08, 0.48)	0.151	0.20	0.21 (− 0.08, 0.49)	0.152	0.20
	Post-treatment	0.22 (− 0.10, 0.54)	0.186	0.20	0.25 (− 0.08, 0.58)	0.135	0.24
	6-month follow-up	0.19 (− 0.13, 0.51)	0.247	0.19	0.17 (− 0.16, 0.49)	0.327	0.15
Mindfulness skills							
FFMQ-SF mindfulness skills	Overall	**5.32 (1.80, 8.85)**	0.003*	**0.45**	**4.08 (0.39, 7.76)**	0.030*	**0.33**
	Post-treatment	**5.70 (1.71, 9.69)**	0.005*	**0.49**	**4.50 (0.35, 8.66)**	0.034*	**0.37**
	6-month follow-up	**4.90 (0.89, 8.91)**	0.017*	**0.42**	3.65 (− 0.49, 7.78)	0.084	0.30
SCS-SF self-compassion	Overall	1.37 (− 0.47, 3.22)	0.145	0.18	0.23 (− 1.60, 2.05)	0.809	0.03
	Post-treatment	1.41 (− 0.84, 3.65)	0.219	0.18	− 0.53 (− 2.67, 1.62)	0.632	− 0.07
	6-month follow-up	1.34 (− 0.92, 3.60)	0.245	0.17	0.97 (− 1.16, 3.11)	0.371	0.14

Bold* indicates significant intervention effect (corrected for multiple subscales); ^ indicates an effect below .05, but not significant after correcting for multiple subscales.

*MBCT* mindfulness-based cognitive therapy; *CRT* cognitive rehabilitation therapy; *ETAU* enhanced treatment as usual; *HADS* Hospital Anxiety and Depression Scale; *CIS-20-* Checklist Individual Strength; *MSQoL-54* Multiple Sclerosis Quality of Life Questionnaire-54; *RRS-NL* Ruminative Response Scale; *MHC-SF* Mental Health Continuum-Short Form; *FFMQ-SF* Five Facets of the Mindfulness Questionnaire Short Form; *SCS-SF* Self-Compassion Scale Short Form; *USER-P* Utrecht Scale for Evaluation of Rehabilitation-Participation

^a^As PROM consisted of two subscales, the α-level of 0.05 was divided by three, resulting in an α-level of 0.025

^b^As PROM consisted of three subscales, the α-level of 0.05 was divided by three, resulting in an α-level of 0.017

### Mediation analyses

**Fig. 3:**
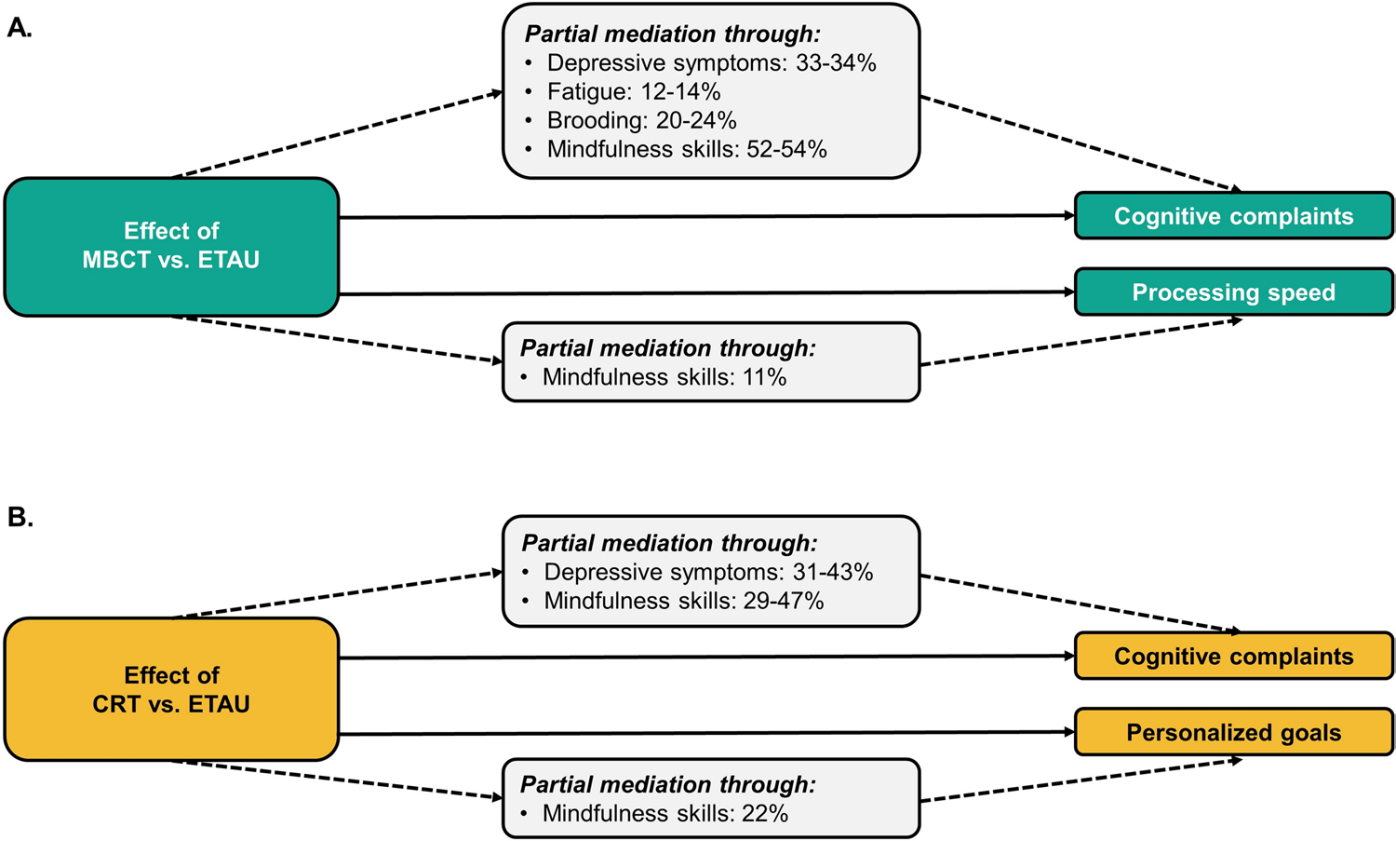
Mediation of psychological symptoms and mindfulness skills on cognitive outcomes. **A** Significant mediators of the effect of MBCT compared to ETAU on self-reported cognitive complaints and information processing speed. **B** Significant mediators of the effect of CRT compared to ETAU on self-reported cognitive complaints and personalized cognitive goals. *CRT* cognitive rehabilitation therapy; *MBCT* mindfulness-based cognitive therapy; *ETAU* enhanced treatment as usual

Significant outcomes for psychological symptoms or mindfulness skills per treatment were subsequently examined as potential mediators (Fig. 3; Supplementary Table 3) of the treatment effects on cognitive outcomes (cognitive outcomes were published previously). Positive effects of MBCT on self-reported executive functioning (BRIEF; metacognition and behavioral regulation) were partially mediated by depressive symptoms (*∆*β = 33.9%, *∆*β = 32.7%, respectively), fatigue (*∆*β = 13.6%, *∆*β = 12.1%, respectively), brooding (*∆*β = 19.9%, *∆*β = 23.5%, respectively), and mindfulness skills (*∆*β = 53.5%, *∆*β = 52.2%, respectively). All these mediators were related to the aforementioned cognitive outcome (*p* < 0.05; except for fatigue on BRIEF behavioral regulation). The positive effect of MBCT on information processing speed at 6-month follow-up was not mediated by depression, fatigue, or brooding (*∆*β < 10%; mediators *p* > 0.05), and although mindfulness skills did seem to reduce the treatment effect (*∆*β = 10.8%), they were not related to information processing speed (*p* = 0.600). Changes in these symptoms between baseline and post-treatment did not mediate this 6-month follow-up effect on information processing speed either (*∆*β < 10%, mediators *p* > 0.05).

As to the positive effect of CRT on self-reported general cognitive complaints (CFQ) and self-reported executive functioning (BRIEF; metacognition) at post-treatment, effects were partly mediated by depression (*∆*β = 43.0%, *∆*β = 30.6%, respectively) and mindfulness skills (*∆*β = 28.9%, *∆*β = 46.6%, respectively). All mediators were related to the cognitive outcomes (all *p* < 0.001). The effect of CRT on personalized cognitive goals at 6-month follow-up was partly mediated by mindfulness skills, both the level of mindfulness skills at 6-month follow-up (*∆*β = 22.1%; mediator *p* < 0.001) and the change between baseline and post-treatment (*∆*β = 28.2%; mediator *p* = 0.038). Depressive symptoms did not mediate this effect of depressive symptoms at 6-month follow-up: *∆*β = 2%, mediator *p* = 0.204; change in depression between baseline and post-treatment: *∆*β = 14.1%, mediator *p* = 0.854).

## Discussion

In this RCT involving MS patients with cognitive complaints, two non-pharmacological interventions, i.e., MBCT and CRT, had a positive effect on psychological symptoms, mindfulness skills, and mental QoL compared to ETAU. The effect on mindfulness skills following MBCT was the only effect still present 6 months later. These effects on psychological symptoms and mindfulness skills mediated the treatment effects found on self-reported cognitive complaints in MS, but not on information processing speed.

Psychological symptoms are common in patients with MS, yet they are rarely targeted in treatment, despite their significant impact on patients’ QoL [7]. This study shows that MBCT reduced a wide variety of self-reported psychological symptoms directly following treatment completion, including depressive symptoms, fatigue and brooding, of which the latter is a central target of mindfulness interventions. These small-to-moderate effects are in accordance with previous mindfulness-based studies [13], but we extend these findings by revealing the feasibility and effectiveness of MBCT within a sample that encompasses cognitively impaired patients. Patients with cognitive impairments are generally not included in previous studies [13], whereas our sample included 57% of these patients. Notably, treatment effects on depressive symptoms [48] and fatigue [49] match the small-to-moderate effect sizes observed in cognitive behavioral therapy (CBT) for MS. CBT is a widely used treatment that typically targets a single psychological symptom, particularly depressive complaints [48]. In contrast to previous studies [50], we did not find a reliable treatment effect of MCBT on anxiety, despite a small effect size (*d* = −0.36, *p* = 0.043). Importantly, MBCT showed moderate-to-large improvements in mental QoL immediately following treatment completion. These improvements are even larger compared to those reported in previous studies [13], highlighting the potential of mindfulness in addressing QoL in MS.

In addition to MBCT, CRT also positively affected depressive symptoms to the same level as MBCT, showing that a treatment primarily focusing on cognitive impairments can also affect psychological symptoms. Previous CRT studies focusing on psychological symptoms in MS are limited, and available results are mixed. For instance, an imagery-based memory training did not reduce psychological symptoms [19]. Conversely, interventions that are more similar to our approach, such as behavioral training with self-generated learning techniques [20] and interventions aimed at increasing awareness of cognitive strengths, limitations, and coping strategies [18], did lead to a reduction in depressive symptoms. Reliable effects of CRT on QoL have also not been established yet, although previous studies do suggest small improvements in QoL [18], positive effects on general contentment in daily life [19, 20], and more confidence in own abilities [51]. Building upon these preliminary findings, our study highlights that depressive symptoms and mental QoL can be moderately improved after compensatory CRT. For both MBCT and CRT, the post-treatment effects of psychological outcomes and QoL subsided 6 months after treatment completion, presumably due to less frequent practice of mindfulness exercises and application of cognitive strategies. A longer lasting intervention or booster sessions may contribute to the consolidation of these treatment effects [52].

We additionally investigated whether the treatment effects transferred to daily-life functioning. However, no significant effects were observed following either treatment. Research on the effects of MBCT and CRT on daily functioning in MS is currently lacking, but it has been argued that improving patient’ everyday life should be an fundamental objective of CRT [17]. Upon closer examination of the used measure (i.e., the USER-P), the lack of effect might be attributed to the scale’s potential inability to detect more subtle changes in patient’s daily life. This questionnaire has primarily been used within rehabilitation settings for patients with physical disabilities [34]. In the current study, average EDSS scores for both treatment groups were below four, indicating the absence of gait difficulties. This may imply that the sample could potentially exceed the scale’s sensitivity in detecting changes following intervention.

In contrast to the previously reported short-term effects on psychological symptoms and QoL, we showed a long-lasting increase of mindfulness skills after MBCT. This finding is promising, as it suggests the persistence of certain effects, although these enhanced mindfulness skills did not seem to transfer to long-term benefits in psychological functioning. Moreover, CRT had a positive effect on mindfulness skills immediately following treatment completion. Even though mindfulness exercises were not included in CRT, the treatment included a session on the handling of emotional and behavioral changes, which may explain this effect. Neither MBCT nor CRT had an impact on general well-being and self-compassion, despite the latter being an important aspect of MBCT. It is possible that self-compassion may require more specific training, as is offered in Mindfulness-Based Compassionate Living [53], to show improvements.

Interestingly, we found that treatment effects on self-reported cognitive complaints and cognitive goals were mediated by the reduction of psychological symptoms and an increase in mindfulness skills, whereas treatment effects on IPS were not mediated by these symptoms. This finding matches the previously reported discrepancy between self-reported cognitive problems versus objectively tested cognitive performance in MS patients, as psychological symptoms mainly relate to self-reported cognitive complaints and not to objective cognitive performance [54–56]. In the current study, we selected patients based on the presence of self-reported cognitive complaints, and indeed, a large proportion of patients also experienced severe fatigue (88%). This high level of fatigue in our sample also indicates that both MBCT and CRT are feasible in severely fatigued patients. Despite the relation between self-reported cognition and depressive symptoms [57], only 10% of patients in our sample experienced severe depressive symptoms. This illustrates that even in patients with below-threshold depressive symptoms, moderate reductions in depressive symptoms can be achieved after MBCT and CRT. It would be clinically relevant to investigate whether MS patients who have a clinical depression also benefit from these treatments. Notably, the reduction of depressive symptoms, combined with increased mindfulness skills, even showed the largest mediating effects (~ 30–50%) on self-reported cognitive function. However, it must be noted that these mediators were analyzed in a cross-sectional manner, and no conclusion regarding causality can be drawn. Regarding the 6-month follow-up effect on cognitive goals, our findings indicate that changes in mindfulness skills directly following CRT completion preceded the long-term benefits on personalized cognitive goals 6 months later. Combined, our results implicate that the effects of MBCT and CRT on self-reported cognitive function and psychological symptoms are interrelated, whereas the effect of MBCT on information processing speed seemed to be independent. The multi-dimensional effect of MBCT and CRT is clinically relevant, given that patients generally engage in multiple treatments at the same time (e.g., DMT, physiotherapy). It thus may be advantageous to target both psychosocial *and* cognitive problems simultaneously.

Several limitations of our study need to be considered. Both our treatments were group based, and we cannot rule out that several effects resulted from non-specific treatment characteristics, such as peer support. Future studies may benefit by including a group-based control condition. Still, the differential effect of MBCT and CRT on various factors (e.g., brooding and fatigue were only affected after MBCT), suggests the presence of treatment-specific effects. Secondly, patients could by definition not be blinded for treatment allocation, which, in combination with the self-reported outcomes, may have resulted in biased results. Another potential source of bias could have been the inclusion of patients with cognitive complaints. While it is promising that MBCT and CRT yield beneficial effects for these patients, it also raises the question to what extent these results generalize to patients without cognitive complaints. Lastly, the power of the REMIND-MS study was based on self-reported cognitive complaints (i.e., primary outcome REMIND-MS study) and not on the other PROMS analyzed in current study. Still, we were able to find both small and large treatments effects, indicating that the sample size was sufficient. It is noteworthy that most of the treatment effects found in the current study were even larger in size than the previously published effects on self-reported cognitive complaints [14].

To conclude, in MS patients with self-reported cognitive complaints, high levels of fatigue, and with more than 50% being cognitively impaired, MBCT reduced a wide array of psychological symptoms, including fatigue, depressive symptoms, and brooding, and led to improved mental QoL. Combined with its previously shown effect on information processing speed, which is the most affected cognitive domain in MS patients, mindfulness leads to significant benefits in MS. Likewise, compensatory CRT resulted in a reduction of depressive symptoms and improved mental QoL in the short-term, indicating that CRT is not solely a cognitive treatment but is also effective in improving psychosocial symptoms. For now, it is crucial to investigate how these beneficial effects can be maintained over a longer period of time. Considering the uncertainty of the MS disease course, combined with the large burden of symptoms and relatively young age of onset, consolidation of treatment effects by continuing support is of the utmost importance.

### Supplementary Information

Below is the link to the electronic supplementary material.

Supplementary file1 (DOCX 25 KB)

## Data Availability

Anonymized data, not published in the article, will be shared upon reasonable request from a qualified investigator.
