# Supplementary material for: Improved quality of life and psychological symptoms following mindfulness and cognitive rehabilitation in multiple sclerosis and their mediating role for cognition: a randomized controlled trial
Source: J Neurol. 2024 Apr 23;271(7):4361–72. doi: 10.1007/s00415-024-12327-y (PMC11233341; doi:10.1007/s00415-024-12327-y)
Supplement: Supplementary file 1 — Supplementary file1 (DOCX 25 KB) [file 415_2024_12327_MOESM1_ESM.docx]

**Supplementary Table 1.** Construction of the cognitive domains in the REMIND-MS study, based on previously publication [1].

| **Cognitive domain** | **Cognitive (sub-)tests** |
| --- | --- |
| Information processing speed | - Symbol Digit Modalities Test (total number of correctly substituted items) - Stroop Color-Word Test (time to complete cards I and II) |
| Memory | - California Verbal Learning Test (immediate recall, long-term recall and long-term recognition hits scores) - Brief Visuospatial Memory Test-Revised (immediate recall, long-term recall and long-term recognition scores) |
| Visuospatial processing | - Benton Judgement of Line Orientation Test (total number of correct items) |
| Executive function | - Controlled Oral Word Association Test (total score of three letters) - Stroop Color-Word Test (interference score; time to complete card III, corrected for card I and II) - Delis-Kaplan Executive Function System sorting test (free sorting condition with the total number of correct sorts) |

**Supplementary Table 2.** Outcomes (raw scores) per group at each time-point**.**

|  | | **Baseline** | | | **Post-treatment** | | | **6-month follow-up** | | |
| --- | --- | --- | --- | --- | --- | --- | --- | --- | --- | --- |
|  | | **MBCT**  ***n*=32** | **CRT**  ***n*=32** | **ETAU**  ***n*=35** | **MBCT**  ***n*=32** | **CRT**  ***n*=32** | **ETAU**  ***n*=34^a^** | **MBCT**  ***n*=32** | **CRT**  ***n*=32** | **ETAU**  ***n*=35** |
| **Psychological symptoms** | | | | | | | | | | |
|  | HADS anxiety | 8.0 ± 4.3 | 7.3 ± 4.1 | 7.9 ± 4.0 | 5.8 ± 4.3 | 5.8 ± 4.1 | 7.3 ± 4.0 | 6.5 ± 3.3 | 6.3 ± 3.9 | 6.7 ± 4.7 |
|  | HADS depression^b^ | 5.0 (3.0-8.0) | 4.0 (3.0-7.0) | 4.0 (3.0-7.0) | 3.0 (2.0-5.0) | 3.0 (1.0-6.0) | 4.5 (3.0-8.0) | 4.0 (2.0-7.0) | 4.0 (1.0-5.0) | 4.0 (2.0-7.0) |
|  | CIS20-R subjective fatigue | 40.8 ± 12.0 | 38.6 ± 10.6 | 38.5 ± 9.3 | 36.1 ± 11.9 | 35.8 ± 10.1 | 38.5 ± 10.0 | 37.7 ± 12.9 | 36.5 ± 10.2 | 37.7 ± 10.3 |
|  | RRS-NL brooding | 9.4 ± 3.1 | 9.3 ± 2.5 | 9.8 ± 3.1 | 8.4 ± 2.3 | 9.2 ± 2.6 | 9.7 ± 3.0 | 8.9 ± 3.4 | 8.7 ± 2.5 | 9.9 ± 2.9 |
| **Quality of life** | | | | | | | | | | |
|  | MSQoL-54 physical | 51.4 ± 17.4 | 52.3 ± 16.3 | 54.3 ± 16.7 | 55.6 ± 16.9 | 56.9 ± 17.0 | 54.5 ± 16.8 | 53.8 ± 18.0 | 54.2 ± 17.7 | 57.6 ± 16.3 |
|  | MSQoL-54 mental^b^ | 61.8 (47.8-78.4) | 71.3 (43.1-81.4) | 66.5 (50.7-76.7) | 76.9 (65.6-85.7) | 76.3 (54.2-86.0) | 66.0 (47.0-76.4) | 66.9 (53.2-78.8) | 77.0 (56.8-84.3) | 71.8 (48.5-81.6) |
| **Well-being** | | | | | | | | | | |
|  | MHC-SF well-being | 2.8 ± 0.9 | 2.9 ± 1.0 | 2.9 ± 0.9 | 3.0 ± 0.9 | 3.2 ± 1.0 | 2.9 ± 1.0 | 2.9 ± 1.0 | 3.1 ± 1.1 | 2.9 ± 1.1 |
|  | FFMQ-SF mind. skills | 79.3 ± 11.7 | 79.9 ± 11.0 | 77.7 ± 11.3 | 85.3 ± 9.9 | 84.8 ± 11.4 | 78.5 ± 12.5 | 84.1 ± 11.8 | 83.8 ± 12.7 | 78.8 ± 11.7 |
|  | SCS-SF self-compassion | 27.6 ± 8.2 | 26.0 ± 7.2 | 24.6 ± 7.0 | 29.5 ± 7.9 | 26.1 ± 7.1 | 25.4 ± 7.5 | 29.2 ± 7.9 | 27.6 ± 6.5 | 25.4 ± 7.3 |
| **Daily life functioning** | | | | | | | | | | |
|  | USER-P frequency | 32.3 ± 10.0 | 32.0 ± 7.2 | 28.7 ± 9.8 | 30.6 ± 10.1 | 31.4 ± 8.9 | 30.6 ± 9.6 | 30.4 ± 9.0 | 32.9 ± 7.7 | 29.0 ± 10.9 |
|  | USER-P restrictions | 70.0 ± 15.9 | 70.9 ± 13.6 | 70.8 ± 17.7 | 71.0 ± 16.4 | 73.3 ± 14.6 | 74.5 ± 18.4 | 72.6 ± 17.3 | 71.1 ± 17.3 | 72.3 ± 18.9 |
|  | USER-P satisfaction | 57.9 ± 18.0 | 62.7 ± 15.1 | 60.9 ± 17.8 | 64.1 ± 15.9 | 63.7 ± 17.0 | 66.0 ± 16.3 | 58.6 ± 16.1 | 65.1 ± 15.1 | 66.4 ± 14.5 |
| **Cognition (self-reported and objective)** | | | | | | | | | | |
|  | CFQ | 50.2 ± 15.3 | 52.6 ± 11.2 | 51.3 ± 14.1 | 43.2 ± 14.3 | 44.3 ± 15.6 | 49.7 ± 13.8 | 42.6 ± 17.6 | 45.1 ± 15.4 | 45.9 ± 13.9 |
|  | BRIEF-A-BR | 51.8 ± 9.3 | 53.1 ± 11.7 | 54.1 ± 9.6 | 47.9 ± 8.8 | 51.5 ± 11.2 | 53.2 ± 10.0 | 49.0 ± 11.1 | 51.3 ± 11.1 | 52.0 ± 11.6 |
|  | BRIEF-A-MC | 76.3 ± 14.4 | 79.4 ± 10.9 | 79.4 ± 13.4 | 70.6 ± 13.4 | 74.3 ± 13.0 | 79.6 ± 14.5 | 72.1 ± 17.6 | 75.6 ± 14.0 | 76.7 ± 15.0 |
|  | IPS | 0.1 ± 1.1 | 0.02 ± 0.5 | -0.1 ± 1.0 | 0.2 ± 0.9 | 0.1 ± 0.6 | -0.2 ± 1.1 | 0.2 ± 0.9 | 0.1 ± 0.6 | -0.2 ± 1.1 |
|  | GAS^c^ | n/a | n/a | n/a | 49.4 ± 9.7 | 49.3 ± 8.4 | 46.1 ± 7.4 | 50.3 ± 9.5 | 51.8 ± 10.3 | 47.8 ± 11.5 |

Displayed are the mean ± standard deviation. ^a^ One patient of the ETAU group did not have post-treatment measurement. ^b^ For not-normally distributed data, median and interquartile range are indicated. ^c^ At baseline, GAS scores are similar for each patient by definition. *Abbreviations: CRT=cognitive rehabilitation therapy; MBCT=mindfulness-based cognitive therapy; ETAU=enhanced treatment as usual; HADS=Hospital Anxiety and Depression Scale; CIS20-R=Checklist Individual Strength-20; RRS-NL=Ruminative Response Scale; MSQoL-54=Multiple Sclerosis Quality of Life Questionnaire-54; MHC-SF=Mental Health Continuum-Short Form; FFMQ-SF mindf. skills=Five Facets of the Mindfulness Questionnaire short form, mindfulness skills; SCS-SF=Self-Compassion Scale short form; USER-P=Utrecht Scale for Evaluation of Rehabilitation – Participation; CFQ=Cognitive Failure Questionnaire; (I-)BRIEF-A = (Informant) Behavior Rating Inventory of Executive Function – Adult Version; BR=behavioral regulation; MC=metacognition; IPS = Information Processing Speed; GAS=Goal Attainment Scaling.*

**Supplementary Table 3.** Mediation effects of patient-reported outcomes on cognition.

|  |  | **MBCT vs. ETAU** | | | | | |
| --- | --- | --- | --- | --- | --- | --- | --- |
|  |  | ***Group effect*** | ***Group effect including mediator*** | | | ***Mediation effect*** | |
| ***Outcome measure*** | ***Time-point*** | ***β (95%CI)*** | ***Mediator*** | ***β (95%CI)*** | ***p*** | ***∆β%^1^*** | ***p mediator*** |
| Cognitive complaints \| EF metacognition (BRIEF-A MC) | Post-treatment | -5.8 (-10.5, -1.2) | Depression | -3.8 (-8.1, 0.5) | .080 | **33.9*** | **<.001*** |
|  |  | -6.4 (-11.0, -1.8) | Fatigue | -5.6 (-10.1, -1.0) | .017 | **13.6*** | **.033*** |
|  |  | -5.6 (-10.3, -0.9) | Brooding | -4.5 (-9.0, 0.01) | .050 | **19.9*** | **<.001*** |
|  |  | -5.5 (-10.2, -0.9) | Mind. skills | -2.6 (-6.7, 1.5) | .218 | **53.5*** | **<.001*** |
| Cognitive complaints \| EF behavioral regulation (BRIEF-A BR) | Post-treatment | -3.7 (-6.9, -0.4) | Depression | -2.5 (-5.6, 0.6) | .120 | **32.7*** | **<.001*** |
|  |  | -4.0 (-7.3, -0.8) | Fatigue | -3.5 (-6.8, -0.3) | .035 | **12.1*** | .097 |
|  |  | -3.6 (-6.9, -0.3) | Brooding | -2.7 (-5.9, 0.4) | .090 | **23.5*** | **.002*** |
|  |  | -3.6 (-6.8, -0.3) | Mind. skills | -1.7 (-4.7, 1.3) | .271 | **52.2*** | **<.001*** |
| Information processing speed | 6-month follow-up | .22 (0.02, 0.4) | Depression | .22 (0.03, 0.4) | .028 | -0.9 | .892 |
|  |  | .24 (0.03, 0.4) | Fatigue | .23 (0.03, 0.4) | .027 | -0.01 | .870 |
|  |  | .23 (0.04, 0.4) | Brooding | .24 (0.04, 0.4) | .016 | -2.7 | .598 |
|  |  | .24 (0.04, 0.4) | Mind. skills | .21 (0.01, 0.4) | .041 | **10.8*** | .600 |
|  |  | **CRT vs. ETAU** | | | | | |
|  |  | ***Group effect*** | ***Group effect including mediator*** | | | ***Mediation effect*** | |
| ***Outcome measure*** | ***Time-point*** | ***β (95%CI)*** | ***Mediator*** | ***β (95%CI)*** | ***p*** | ***∆β%^1^*** | ***p mediator*** |
| Cognitive complaints \| General (CFQ) | Post-treatment | -5.7 (-9.8, -1.6) | Depression | -3.2 (-7.1, 0.6) | .097 | **43.0*** | **<.001*** |
|  |  | -5.9 (-10.3, -1.5) | Mind. skills | -4.2 (-8.4, -0.05) | .047 | **28.9*** | **<.001*** |
| Cognitive complaints \| EF metacognition (BRIEF-A MC) | Post-treatment | -4.8 (-8.3, -1.2) | Depression | -3.3 (-6.7, 0.1) | .055 | **30.6*** | **<.001*** |
|  |  | -4.9 (-8.8, -1.1) | Mind. skills | -2.6 (-5.9, 0.7) | .116 | **46.6*** | **<.001*** |
| Personalized cognitive goals (GAS) | 6-month follow-up | 4.2 (0.1, 8.3) | Depression | 4.1 (0.02, 8.2) | .049 | 2.4 | .204 |
|  |  | 4.2 (-0.1, 8.5) | Mind. skills | 3.3 (-0.09, 7.4) | .123 | **22.1*** | **<.001*** |

*Note.* ^1^Percentage of change in group effect (β) when including the mediator compared to the model without the mediator. *∆β>10%, which indicates a mediation effect if the mediator also relates to the cognitive outcome (*p* mediator <.05). All models include baseline outcome, baseline mediator, age, education and sex as covariates. *Abbreviations: CRT=cognitive rehabilitation therapy; MBCT=mindfulness-based cognitive therapy; ETAU=enhanced treatment as usual; CFQ=Cognitive Failure Questionnaire; EF = executive function; BRIEF-A= Behavior Rating Inventory of Executive Function – Adult Version; BR=behavioral regulation; MC=metacognition; GAS=Goal Attainment Scaling.*

**Supplemental references**

[1] I. M. Nauta *et al.*, "Cognitive rehabilitation and mindfulness reduce cognitive complaints in multiple sclerosis (REMIND-MS): a randomized controlled trial," *Multiple Sclerosis and Related Disorders,* p. 104529, 2023.
